# Gene Expressions for Signal Transduction under Acidic Conditions

**DOI:** 10.3390/genes4010065

**Published:** 2013-03-08

**Authors:** Toshihiko Fukamachi, Syunsuke Ikeda, Xin Wang, Hiromi Saito, Masatoshi Tagawa, Hiroshi Kobayashi

**Affiliations:** 1 Graduate School of Pharmaceutical Sciences, Chiba University, 1-8-1, Inohana, Chuo-ku, Chiba 260-8675, Japan; E-Mails: t.fukamachi2011@gmail.com (T.F.); seika-admin@p.chiba-u.ac.jp (S.I.); oukin@chiba-u.jp (X.W.); hiromi.saito@thu.ac.jp (H.S.); 2 Division of Pathology and Cell Therapy, Chiba Cancer Center Research Institute, 666-2, Nitona, Chuo-ku, Chiba 260-8717, Japan; E-Mail: mtagawa@chiba-cc.jp

**Keywords:** gene expression, acidic conditions, signal pathways, cancer cells

## Abstract

Although it is now well known that some diseased areas, such as cancer nests, inflammation loci, and infarction areas, are acidified, little is known about cellular signal transduction, gene expression, and cellular functions under acidic conditions. Our group showed that different signal proteins were activated under acidic conditions compared with those observed in a typical medium of around pH 7.4 that has been used until now. Investigations of gene expression under acidic conditions may be crucial to our understanding of signal transduction in acidic diseased areas. In this study, we investigated gene expression in mesothelioma cells cultured at an acidic pH using a DNA microarray technique. After 24 h culture at pH 6.7, expressions of 379 genes were increased more than twofold compared with those in cells cultured at pH 7.5. Genes encoding receptors, signal proteins including transcription factors, and cytokines including growth factors numbered 35, 32, and 17 among the 379 genes, respectively. Since the functions of 78 genes are unknown, it can be argued that cells may have other genes for signaling under acidic conditions. The expressions of 37 of the 379 genes were observed to increase after as little as 2 h. After 24 h culture at pH 6.7, expressions of 412 genes were repressed more than twofold compared with those in cells cultured at pH 7.5, and the 412 genes contained 35, 76, and 7 genes encoding receptors, signal proteins including transcription factors, and cytokines including growth factors, respectively. These results suggest that the signal pathways in acidic diseased areas are different, at least in part, from those examined with cells cultured at a pH of around 7.4.

## 1. Introduction

In mammals, the pH values of blood and tissues are usually maintained in a narrow range around 7.4 [1]. In contrast, diseased areas, such as cancer nests, inflammatory loci, and infarction areas, have been found to be acidic. The extracellular pH in the central regions of tumors decreases below 6.7 in several tumors as a consequence of lactate accumulation derived from a lack of sufficient vascularization or an increase in tumor-specific glycolysis under aerobic conditions combined with impaired mitochondrial oxidative phosphorylation [1,2,3]. Extracellular pH may also drop to a value below 6 due to leaking of intracellular contents and the destruction of blood vessels resulting in hypoxic metabolism and related lactic acid production during inflammation against the infection of pathogens [4]. Similar acidic environments were also associated with other inflammation. The pH value of articular fluid in the rheumatoid human knee joint was around 6.6, compared to around 7.3 in normal knee joints [5]. Other studies also showed the acidification of synovial fluid in arthritis [6,7,8].

Although cell functions mediated by a large number of enzymes with pH-dependent catalytic activity are strongly affected by the disruption of pH homeostasis, there have been only a few studies of signal transduction, gene expression, and cellular functions under acidic conditions. Studies of *Escherichia coli* have suggested that this bacterium has multiple systems for a single function and that different systems having optimum activities at different pH values function under different pH conditions [9,10].

Our group previously found that different signal transduction pathways function under acidic environments [11,12], and that CTIB, an IκB-β variant, acted as a critical factor at pH 6.3 but not at pH 7.4 [13,14]. Our group also showed the elevated activation of p38 and ERK in human T cells cultured at acidic pH [12,15]. In addition to these reports by our group, activation of the MAPK pathways and increased COX-2 protein expression were reported in acid exposed cells in Barrett’s metaplasia [16]. Matrix metalloproteinase-9 (MMP-9) expression was induced at acidic extracellular pH in mouse metastatic melanoma cells through phospholipase D-mitogen-activated protein kinase signaling [17]. Carbonic anhydrase 9 (CA9) expression was increased by acidosis via a hypoxia-independent mechanism that operates through modulation of the basic CA9 transcriptional machinery [18]. The gene expression of VEGF was stimulated at low extracellular pH [19,20]. Glioma stem cells grown in low pH conditions displayed an increase in expressions of Olig2, Oct4, Nanog, interleukin-8 (IL-8), TIMP1, TIMP2, VEGF, Glut1, SerpinB9, and HIF2α, whereas expressions of Sox2, GFAP, and HIF1α were repressed in the cells [21]. The expression of HIF1α induced by hypoxia was decreased by acidosis and the expression of ATF4 was increased by the combination of acidosis with hypoxia [22].

These previous findings led us to assume that different signal pathways operate under acidic conditions in mammalian cells. In addition to the molecules reported in previous studies described above, numerous molecules may work preferentially under low pH conditions. To exhaustively identify genes working for cell proliferation under acidic conditions, we used cancer cells that were able to proliferate rapidly and investigated the gene expression in mesothelioma cells cultured at acidic pH using a DNA microarray technique in the present study. After 24 h culture at pH 6.7, expressions of 379 genes were increased more than twofold compared with those in cells cultured at pH 7.5. The 379 genes contained 84 genes encoding receptors, signal proteins, transcription factors, cytokines, and growth factors, suggesting that the signal pathways in acidic diseased areas are different, at least in part, from those examined with cells cultured at pH around 7.4. The identified genes may be potential candidates for cancer chemotherapeutics. After 24 h culture at pH 6.7, expressions of 412 genes were repressed more than twofold compared with those in cells cultured at pH 7.5, and genes encoding receptors, signal proteins, transcription factors, cytokines, and growth factors numbered 118 among the 412 genes.

## 2. Materials and Methods

### 2.1. Cells and Medium for Their Maintenance

Human mesothelial cell line H2052, human colon adenocarcinoma grade II cell line HT-29, human esophageal cancer cell line TE-11, human pancreatic ductal adenocarcinoma cell line BxPC3, and human hepatocellular carcinoma cell line HepG2 were used. For cell maintenance, cells were cultured in RPMI-1640 (WAKO) containing 10 μg/mL gentamicin (Sigma), 1 μg/mL fungizone (Bristol-Myers), and 10% FBS (Sigma) in the presence of 5% CO_2_ at 37 °C.

### 2.2. Cell Culture under Different pH Conditions

Media for cell culture at various pH values were prepared as follows. To minimize the pH change during the cell culture, 10 mM PIPES [piperazine-*N*,*N*'-bis(ethanesulfonic acid)] for acidic media or HEPES [4-(2-hydroxyethyl)piperazine-1-ethanesulfonic acid] for alkaline media was added to RPMI-1640 instead of NaHCO_3_. Medium containing fetal bovine serum (FBS) was often contaminated with germs when the medium pH was adjusted, and it was hard to sterilize medium containing FBS. Therefore, medium pH was first adjusted by the addition of NaOH to 6.3 and 7.6 before the addition of FBS. After sterilization of the medium by filtration, FBS was added. The medium pH values were changed into 6.7 and 7.5 by the addition of FBS, respectively. Cells were cultured at 37 °C without a CO_2_ supply but with an air supply to avoid hypoxia and constant humidity.

### 2.3. DNA Microarray

After H2052 cells had been cultured in pH 7.5 medium as described above for 24 h at 37 °C, the medium was exchanged for pH 6.7 medium, and cells were cultured at 37 °C for 2, 5, and 24 h. Total RNA was isolated with the use of a TRI reagent (Sigma) according to the manufacturer’s instructions, and microarray analysis was entrusted to Roche Diagnostics Corporation using the Roche NimbleGen Microarray A4487001-00-01. In order to compare, data were processed using the NimbleScan software that was developed based on previous papers [23,24].

### 2.4. Real-Time Quantitative Polymerase Chain Reaction (PCR)

Total RNA (1 µg) prepared as described above was reverse-transcribed using ReverTra Ace (TOYOBO) in a total volume of 20 µL containing the random primer for 18S rRNA or the polyT primer for targeted genes. Real-time quantitative PCR amplification was performed with an ABI Prism 7000 Sequence Detection System (Applied Biosystems) using the FastStart Universal SYBR Green Master[Rox] (Roche Diagnostics) according to the manufacturer’s instructions. The PCR reaction was carried out with a mixture containing 12.5 µL of Real-Time PCR Master Mix, 7.5 µM of each sense and antisense primer, 25 ng of cDNA, and nuclease-free water in a total volume of 25 µL. The standard thermal profile for PCR amplification was 50 °C for 2 min, 95 °C for 10 min, and 40 cycles of 95 °C for 15 s and 60 °C for 60 s. The primers used are shown in Table 1.

It has been reported that the content of ribosomes per cell is approximately 4 × 10^6^ [25], and the amount of mRNA per cell can be estimated using 18S rRNA as a control RNA with the following equation.

4 × 10^6^ × 2^{(Ct of 18S rRNA) − (Ct of sample RNA)}^

where Ct is the threshold cycle number.

**Table 1:** Primers used in this study.

Gene name	Sequence	
18S rRNA	F;	TAGAGTGTTCAAAGCAGGCCC
	R;	CCAACAAATAGAACCGCGGT
IL-32	F;	TCAAAGAGGGCTACCTGGAG
	R;	TTTCAAGTAGAGGAGTGAGCTCTG
ATP6V0D2	F;	GACCCAGCAAGACTATATCAACC
	R;	TGGAGATGAATTTTCAGGTCTTC
TNFRSF9	F;	AAACGGGGCAGAAAGAAACT
	R;	CTTCTGGAAATCGGCAGCTA
AREG	F;	GGGAGTGAGATTTCCCCTGT
	R;	AGCCAGGTATTTGTGGTTCG
DMGDH	F;	GAGCTCACGGCTGGATCTAC
	R;	CCACCACCTGACCAGTTTCT
ERBB3	F;	TGCAGTGGATTCGAGAAGTG
	R;	GGCAAACTTCCCATCGTAGA

18S rRNA, 18S ribosomal ribonucleic acid; IL-32, interleukin 32; ATP6V0D2, V0 subunit d2 of lysosomal H^+^ transporting ATPase; TNFRSF9, tumor necrosis factor receptor superfamily member 9; AREG, amphiregulin; DMGDH, dimethylglycine dehydrogenase; ERBB3, erythroblastic leukemia viral oncogene homolog 3.

### 2.5. Other Reagents

Taq DNA polymerase (Bio Academia) and Ribonuclease inhibitor (TOYOBO) were used.

### 2.6. Statistical Analysis

The Student’s t-test was utilized in this study.

## 3. Results

### 3.1. Highly Expressed Genes under Acidic Conditions in Mesothelioma Cells

**Figure 1:**
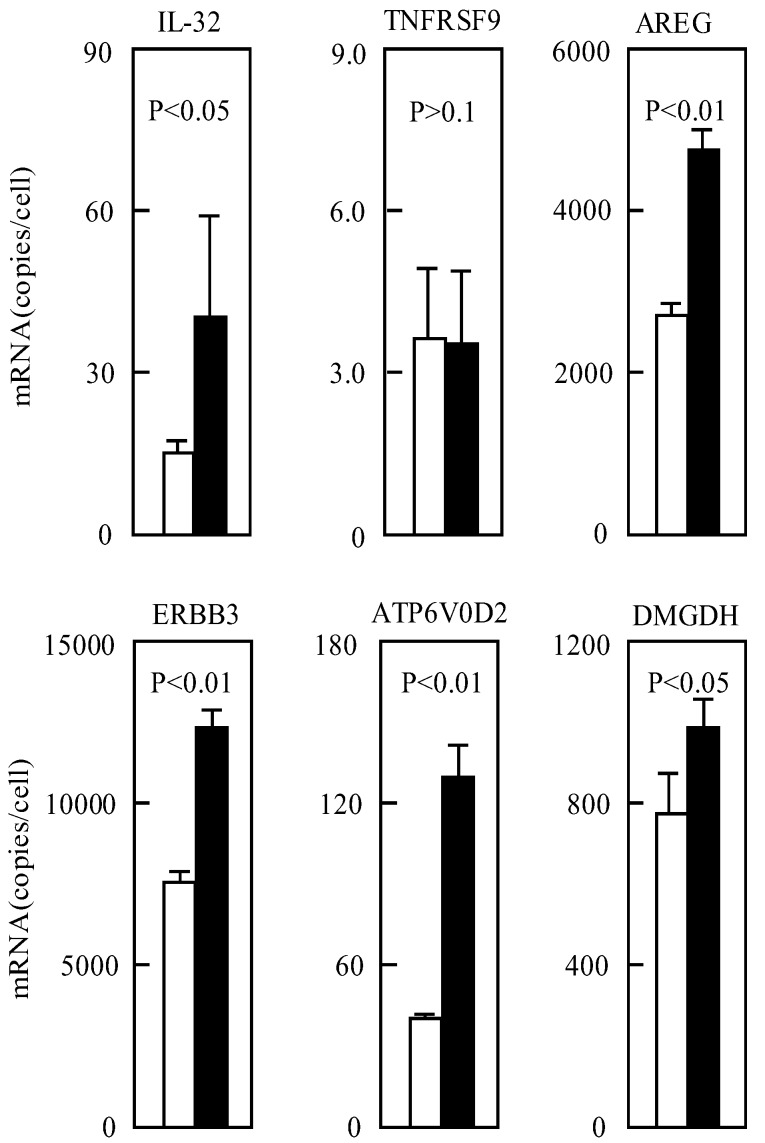
Gene expressions of IL-32, TNFRSF9, AREG, ERBB3, ATP6V0D2, and DMGDH at pH 7.5 and 6.7 in TE-11 cells. TE-11 cells were incubated at pH 7.5 (open bars) and 6.7 (closed bars) for 24 h. mRNA number per one cell was detected with real-time quantitative PCR. Calculation is described in Materials and Methods. The mean values and standard deviations obtained from three independent experiments are represented. P values were calculated as described in Materials and Methods.

Approximately 24,000 genes were examined by microarray in mesothelioma cells (supplementary table), and the expressions of 379 genes were elevated more than twofold in cells cultured at pH 6.7 for 24 h compared with the cells cultured at pH 7.5 (Table 2). The accuracy of microarray analysis is mainly dependent on the RNA preparation. When the copy number of mRNA was low, the standard deviations of real-time quantitative PCR were close to 50% (Figure 1). We therefore assumed that more than twofold changes were significant in the present study. The 379 genes contained 35, 32, and 17 genes encoding receptors, signal proteins including transcription factors, and cytokines including growth factors, respectively (Table 2). The functions of 78 genes among the 379 genes are unknown.

**Table 2:** Genes whose expression was induced more than twofold after 2, 5, and 24 h culture at acidic pH.

Gene	2 h	5 h	24 h
number of genes	260	175	379
receptors	29	22	35
signal proteins ^1^	25	21	32
cytokines ^2^	5	10	17

^1^ including transcription factors; ^2^ including growth factors.

The expressions of IL-8 [21], MMP-9 [17], VEGF [19,20,21], CA9 [18], and COX-2 [16] were reported to increase under acidic stress. Our present results showed that the ratios of the expressions of IL-8, MMP-9, and VEGF in cells cultured at pH 6.7 to those at pH 7.5 were 2.52, 1.90, and 1.12, respectively. The expression of CA9 increased 1.35-fold at pH 6.7, but COX-2 expression was decreased at pH 6.7. It was reported that MnSOD participates in metastasis [26], and our data showed that the increase in the expression of MnSOD at acidic pH was 1.70-fold.

The previous reports by our group showed that p38 and ERK were activated more strongly at acidic pH than at alkaline pH [12,15]. The present data showed that p38-α (MAPK14) expression increased 1.71-fold after 5 h culture at pH 6.7, but decreased after 24 h culture at pH 6.7 (supplementary table), suggesting that p38-α is up-regulated for a short time after cells have been stressed by acidosis. The expressions of p38-γ (MAPK12) and ERK1 increased only 1.29-fold at pH 6.7. The expressions of other p38 and ERK families decreased slightly at acidic pH.

### 3.2. Gene Expression after Culture for a Short Period at pH 6.7

The gene expressions were also examined after 2 and 5 h at pH 6.7, and genes whose expression was increased were classified into seven groups as shown in Table 3. The expressions of 260 genes increased more than twofold in cells cultured at pH 6.7 for 2 h compared with pH 7.5. The 260 genes contained 29, 25, and 5 genes encoding receptors, signal proteins including transcription factors, and cytokines including growth factors, respectively (Table 2). The expressions of 15 among the 260 genes maintained high levels more than twofold for 24 h (Table 3, group A), while the expressions of 223 among the 260 genes decreased again after 24 h (Table 3, groups E and G). The 191 genes were expressed at a high level only at 2 h after the pH shift to 6.7 (Table 3, group G). After 5 h culture at pH 6.7, 175 genes were expressed more than twofold higher than the expression levels at pH 7.5 (Table 2), and the 91 genes were expressed at a high level only at 5 h after acidic stress (Table 3, group F). Genes encoding proteins for signal pathways among the genes whose expression was increased at acidic pH are listed in Table 4.

**Table 3:** Classification of genes whose expression was induced at acidic pH.

Group	Expression level *			Number of genes	
	2 h	5 h	24 h	Total	Signal **
A	>2	>2	>2	15	7
B	>2	<=2	>2	22	3
C	<=2	>2	>2	37	8
D	<=2	<=2	>2	305	66
E	>2	>2	<=2	32	6
F	<=2	>2	<=2	91	32
G	>2	<=2	<=2	191	43
			total	693	165

* ratio of the expression in cells cultured at pH 6.7 to those at pH 7.5; ** genes encoding receptors, signal proteins, transcription factors, cytokines, and growth factors.

**Table 4:** Genes encoding receptors, signal proteins, transcription factors, cytokines, and growth factors whose expression was high at pH 6.7.

Gene	Ratio *	Accession number	Description
**Group A**			
RSPO3	7.346	NM_032784	R-spondin 3 homolog (*Xenopus laevis*)
IL32	3.711	NM_001012631	interleukin 32
TAS2R39	3.035	NM_176881	taste receptor, type 2, member 39
SLAMF8	2.751	NM_020125	SLAM family member 8
TRAF1	2.644	NM_005658	TNF receptor-associated factor 1
IL8	2.519	NM_000584	interleukin 8
RAB33A	2.356	NM_004794	RAB33A, member RAS oncogene family
**Group B**			
LOC553158	4.306	NM_181334	PRR5-ARHGAP8 fusion
PPP1R3E	3.702	XM_927029	protein phosphatase 1, regulatory (inhibitor) subunit 3E
BDKRB2	2.168	NM_000623	bradykinin receptor B2
**Group C**			
TNFRSF9	5.464	NM_001561	tumor necrosis factor receptor superfamily, member 9
FGF7	3.219	NM_002009	fibroblast growth factor 7 (keratinocyte growth factor)
ZNF226	2.926	NM_015919	zinc finger protein 226
MGC17330	2.755	NM_052880	HGFL gene
IL1RAP	2.551	NM_134470	interleukin 1 receptor accessory protein
NFKBIZ	2.159	NM_001005474	nuclear factor of κ light polypeptide gene enhancer in B-cells inhibitor, ζ
OLR1	2.054	NM_002543	oxidized low density lipoprotein (lectin-like) receptor 1
TRIB3	2.031	NM_021158	tribbles homolog 3 (Drosophila)
**Group D**			
ERBB3	5.997	NM_001982	v-erb-b2 erythroblastic leukemia viral oncogene homolog 3 (avian)
AREG	5.650	NM_001657	amphiregulin (schwannoma-derived growth factor)
LOC653193	4.485	XM_926448	similar to Amphiregulin precursor (AR) (Colorectum cell-derived growth factor) (CRDGF)
RARRES1	3.882	NM_002888	retinoic acid receptor responder (tazarotene induced) 1
RRAD	3.827	NM_004165	Ras-related associated with diabetes
CRELD1	3.707	NM_001031717	cysteine-rich with EGF-like domains 1
ARHGAP8	3.547	NM_001017526	Rho GTPase activating protein 8
GPR78	3.302	NM_080819	G protein-coupled receptor 78
GDF15	3.112	NM_004864	growth differentiation factor 15
PTP4A3	3.037	NM_007079	protein tyrosine phosphatase type IVA, member 3
IL16	2.926	NM_004513	interleukin 16 (lymphocyte chemoattractant factor)
PAQR6	2.919	NM_198406	progestin and adipoQ receptor family member VI
OR52N4	2.915	NM_001005175	olfactory receptor, family 52, subfamily N, member 4
OR56B1	2.913	NM_001005180	olfactory receptor, family 56, subfamily B, member 1
PTPRQ	2.834	XM_926134	protein tyrosine phosphatase, receptor type, Q
LOC439957	2.784	XM_495805	similar to Ig κ chain V-I region Walker precursor
TNFSF9	2.744	NM_003811	tumor necrosis factor (ligand) superfamily, member 9
TNFSF7	2.714	NM_001252	tumor necrosis factor (ligand) superfamily, member 7
GPR87	2.641	NM_023915	G protein-coupled receptor 87
**Group D**			
GTF2IRD2B	2.609	NM_001003795	general transcription factor 21 repeat domain containing 2β
RGS7	2.573	NM_002924	regulator of G-protein signalling 7
FOLR3	2.506	NM_000804	folate receptor 3 (γ)
RELB	2.471	NM_006509	v-rel reticuloendotheliosis viral oncogene homolog B, nuclear factor of κ light polypeptide gene enhancer in B-cells 3 (avian)
TAS2R40	2.459	NM_176882	taste receptor, type 2, member 40
CCL3L3	2.418	NM_001001437	chemokine (C-C motif) ligand 3-like 3
GPR144	2.391	NM_182611	G protein-coupled receptor 144
RND1	2.389	NM_014470	Rho family GTPase 1
CD6	2.381	NM_006725	CD6 molecule
ZNF165	2.368	NM_003447	zinc finger protein 165
ICHTHYIN	2.353	XM_371777	ichthyin protein
PKD1L1	2.334	NM_138295	polycystic kidney disease 1 like 1
NPHP1	2.318	NM_207181	nephronophthisis 1 (juvenile)
PTK6	2.312	NM_005975	PTK6 protein tyrosine kinase 6
IL15RA	2.282	NM_002189	interleukin 15 receptor, α
POU6F1	2.271	NM_002702	POU domain, class 6, transcription factor 1
TNFRSF10C	2.268	NM_003841	tumor necrosis factor receptor superfamily, member 10c, decoy without an intracellular domain
IL15	2.248	NM_172175	interleukin 15
P2RY12	2.233	NM_176876	purinergic receptor P2Y, G-protein coupled, 12
MST1	2.186	NM_020998	macrophage stimulating 1 (hepatocyte growth factor-like)
KDR	2.184	NM_002253	kinase insert domain receptor (a type III receptor tyrosine kinase)
GPR68	2.174	NM_003485	G protein-coupled receptor 68
GPR44	2.170	NM_004778	G protein-coupled receptor 44
RAI17	2.162	NM_020338	retinoic acid induced 17
OR10V1	2.156	NM_001005324	olfactory receptor, family 10, subfamily V, member 1
ASB1	2.148	NM_016114	ankyrin repeat and SOCS box-containing 1
CMTM1	2.146	NM_181293	CKLF-like MARVEL transmembrane domain containing 1
PHF7	2.141	NM_173341	PHD finger protein 7
GPRC5D	2.114	NM_018654	G protein-coupled receptor, family C, group 5, member D
TP53INP2	2.108	NM_021202	tumor protein p53 inducible nuclear protein 2
ARHGAP15	2.082	NM_018460	Rho GTPase activating protein 15
GEFT	2.066	NM_182947	RAC/CDC42 exchange factor
PIM1	2.062	NM_002648	pim-1 oncogene
TNFRSF25	2.055	NM_148973	tumor necrosis factor receptor superfamily, member 25
GPR157	2.049	NM_024980	G protein-coupled receptor 157
NR2E3	2.045	NM_014249	nuclear receptor subfamily 2, group E, member 3
LOC619207	2.042	XM_927510	scavenger receptor protein family member
WISP3	2.033	NM_003880	WNT1 inducible signaling pathway protein 3
P2RX4	2.030	NM_002560	purinergic receptor P2X, ligand-gated ion channel, 4
RASD2	2.029	NM_014310	RASD family, member 2
FGF2	2.028	NM_002006	fibroblast growth factor 2 (basic)
RGR	2.011	NM_001012720	retinal G protein coupled receptor
**Group D**			
NRXN2	2.011	NM_015080	neurexin 2
EDG4	2.009	NM_004720	endothelial differentiation, lysophosphatidic acid G-protein-coupled receptor, 4
KGFLP1	2.006	NM_174950	keratinocyte growth factor-like protein 1
PTPRH	2.005	NM_002842	protein tyrosine phosphatase, receptor type, H
OR52A5	2.001	NM_001005160	olfactory receptor, family 52, subfamily A, member 5
**Group E**			
KLF9	3.169	NM_001206	Kruppel-like factor 9
CLASP2	2.029	NM_015097	cytoplasmic linker associated protein 2
E2F5	2.267	NM_001951	E2F transcription factor 5, p130-binding
ZNF474	2.203	NM_207317	zinc finger protein 474
GPR37	2.411	NM_005302	G protein-coupled receptor 37 (endothelin receptor type B-like)
PAX5	2.097	NM_016734	paired box gene 5 (B-cell lineage specific activator)
**Group F**			
OR4D6	2.809	NM_001004708	olfactory receptor, family 4, subfamily D, member 6
OR5B12	2.783	NM_001004733	olfactory receptor, family 5, subfamily B, member 12
VENTX	2.515	NM_014468	VENT homeobox homolog (*Xenopus laevis*)
UNC5B	2.438	NM_170744	unc-5 homolog B (*C. elegans*)
OR1J4	2.416	NM_001004452	olfactory receptor, family 1, subfamily J, member 4
NR5A1	2.338	NM_004959	nuclear receptor subfamily 5, group A, member 1
SESN2	2.331	NM_031459	sestrin 2
CCL25	2.308	NM_148888	chemokine (C-C motif) ligand 25
IL21R	2.301	NM_021798	interleukin 21 receptor
ATF3	2.220	NM_001030287	activating transcription factor 3
TLR1	2.212	NM_003263	toll-like receptor 1
C1QTNF7	2.202	NM_031911	C1q and tumor necrosis factor related protein 7
TBX19	2.186	NM_005149	T-box 19
MXD1	2.163	NM_002357	MAX dimerization protein 1
GTPBP2	2.148	NM_019096	GTP binding protein 2
NR4A2	2.117	NM_006186	nuclear receptor subfamily 4, group A, member 2
PHLDA1	2.116	NM_007350	pleckstrin homology-like domain, family A, member 1
LCP1	2.111	NM_002298	lymphocyte cytosolic protein 1 (L-plastin)
FGFBP1	2.111	NM_005130	fibroblast growth factor binding protein 1
OR2B11	2.078	NM_001004492	olfactory receptor, family 2, subfamily B, member 11
OR56A3	2.073	NM_001003443	olfactory receptor, family 56, subfamily A, member 3
GH2	2.071	NM_002059	growth hormone 2
PTHLH	2.061	NM_002820	parathyroid hormone-like hormone
THBD	2.059	NM_000361	thrombomodulin
HGF	2.055	NM_000601	hepatocyte growth factor (hepapoietin A; scatter factor)
ARTN	2.051	NM_003976	artemin
EPHA8	2.040	NM_020526	EPH receptor A8
CD200R1	2.023	NM_138939	CD200 receptor 1
FZD7	2.017	NM_003507	frizzled homolog 7 (Drosophila)
S100A12	2.012	NM_005621	S100 calcium binding protein A12 (calgranulin C)
**Group F**			
SPIC	2.008	NM_152323	Spi-C transcription factor (Spi-1/PU.1 related)
VSIG4	2.006	NM_007268	V-set and immunoglobulin domain containing 4
Group G			
TCF21	3.518	NM_198392	transcription factor 21
DOK6	2.827	NM_152721	docking protein 6
FZD3	2.815	NM_017412	frizzled homolog 3 (Drosophila)
CD86	2.686	NM_006889	CD86 molecule
OR2L13	2.677	NM_175911	olfactory receptor, family 2, subfamily L, member 13
IL18RAP	2.557	NM_003853	interleukin 18 receptor accessory protein
TRPA1	2.427	NM_007332	transient receptor potential cation channel, subfamily A, member 1
RTP3	2.417	NM_031440	receptor transporter protein 3
GRIA2	2.353	NM_000826	glutamate receptor, ionotropic, AMPA 2
OR51E1	2.351	NM_152430	olfactory receptor, family 51, subfamily E, member 1
GRM2	2.343	NM_000839	glutamate receptor, metabotropic 2
FCRLM1	2.335	NM_032738	Fc receptor-like and mucin-like 1
NR4A3	2.332	NM_173199	nuclear receptor subfamily 4, group A, member 3
SPI1	2.288	NM_003120	spleen focus forming virus (SFFV) proviral integration oncogene spi1
SMAD6	2.282	NM_005585	SMAD, mothers against DPP homolog 6 (Drosophila)
LOC642400	2.281	XM_925921	similar to tripartite motif protein 17
CAMTA1	2.274	NM_015215	calmodulin binding transcription activator 1
GDF6	2.260	NM_001001557	growth differentiation factor 6
OR51B2	2.258	NM_033180	olfactory receptor, family 51, subfamily B, member 2
OR7D4	2.238	NM_001005191	olfactory receptor, family 7, subfamily D, member 4
ECGF1	2.227	NM_001953	endothelial cell growth factor 1 (platelet-derived)
LAIR1	2.218	NM_002287	leukocyte-associated immunoglobulin-like receptor 1
NELL1	2.209	NM_006157	NEL-like 1 (chicken)
OR8J1	2.188	NM_001005205	olfactory receptor, family 8, subfamily J, member 1
GRM3	2.178	NM_000840	glutamate receptor, metabotropic 3
PRKCQ	2.170	NM_006257	protein kinase C, θ
PPP1R3F	2.164	NM_033215	protein phosphatase 1, regulatory (inhibitor) subunit 3F
LOC642338	2.150	XM_925874	similar to vomeronasal 1 receptor, C3
CPNE5	2.148	NM_020939	copine V
EPHB6	2.134	NM_004445	EPH receptor B6
OR51M1	2.119	NM_001004756	olfactory receptor, family 51, subfamily M, member 1
PTPRC	2.105	NM_002838	protein tyrosine phosphatase, receptor type, C
EBF3	2.100	NM_001005463	early B-cell factor 3
BMPR1B	2.091	NM_001203	bone morphogenetic protein receptor, type IB
HRH4	2.084	NM_021624	histamine receptor H4
SHE	2.082	NM_001010846	Src homology 2 domain containing E
T2R55	2.073	NM_181429	taste receptor T2R55
SBK1	2.067	NM_001024401	SH3-binding domain kinase 1
RASGRP2	2.048	NM_005825	RAS guanyl releasing protein 2 (calcium and DAG-regulated)
CD96	2.047	NM_005816	CD96 molecule
GRIN2B	2.028	NM_000834	glutamate receptor, ionotropic, *N*-methyl D-aspartate 2B
**Group G**			
YAF2	2.018	NM_001012424	YY1 associated factor 2
LCP2	2.001	NM_005565	lymphocyte cytosolic protein 2 (SH2 domain containing leukocyte protein of 76 kDa)

* ratio of the expression in cells cultured at pH 6.7 after 24, 5, and 2 h culture to those at pH 7.5 in groups A to D, E to F, and G, respectively.

### 3.3. Genes Whose Expression Was Repressed at Acidic pH

The expressions of 412 genes were repressed more than twofold in cells cultured at pH 6.7 for 24 h, and the 412 genes contained 35, 76, and 7 genes encoding receptors, signal proteins including transcription factors, and cytokines including growth factors, respectively (Table 5). Genes whose expression was repressed at acidic pH were classified into seven groups, as shown in Table 6. The expressions of 17 genes decreased already after 2 h culture at pH 6.7 (Table 6, groups A and B).

The expressions of 385 genes were repressed more than twofold in cells cultured at pH 6.7 for 2 h (Table 5), but the expressions of 368 of these 385 genes increased again after 24 h culture (Table 6, groups E and G). The expressions of 343 among 385 genes were repressed only after 2 h culture at pH 6.7 (Table 6, group G). After 5 h culture at pH 6.7, the expressions of 141 genes were repressed (Table 5) and 76 genes were repressed only after 5 h culture at pH 6.7 (Table 6, group F). Genes encoding proteins for signal pathways among the genes whose expression was repressed at acidic pH are listed in Table 7.

**Table 5:** Genes whose expression was repressed more than twofold after 2, 5, and 24 h culture at acidic pH.

Gene	2 h	5 h	24 h
number of genes	385	141	412
receptors	32	14	35
signal proteins ^1^	31	14	76
cytokines ^2^	8	0	7

^1^ including transcription factors; ^2^ including growth factors.

**Table 6:** Classification of genes whose expression was repressed at acidic pH.

Group	Expression level *			Number of genes	
	2 h	5 h	24 h	Total	Signal **
A	<0.5	<0.5	<0.5	8	4
B	<0.5	>=0.5	<0.5	9	3
C	>=0.5	<0.5	<0.5	32	4
D	>=0.5	>=0.5	<0.5	363	107
E	<0.5	<0.5	>=0.5	25	8
F	>=0.5	<0.5	>=0.5	76	12
G	<0.5	>=0.5	>=0.5	343	56
			total	856	194

* ratio of the expression in cells cultured at pH 6.7 to those at pH 7.5; ** genes encoding receptors, signal proteins, transcription factors, cytokines, and growth factors.

**Table 7:** Genes encoding receptors, signal proteins, transcription factors, cytokines, and growth factors whose expression was repressed at pH 6.7.

Gene	Ratio *	Accession number	Description
**Group A**			
IL11	0.158	NM_000641	interleukin 11
CCRL2	0.324	NM_003965	chemokine (C-C motif) receptor-like 2
CD300LG	0.444	NM_145273	CD300 molecule-like family member g
ATOH1	0.459	NM_005172	atonal homolog 1 (Drosophila)
**Group B**			
RASGEF1C	0.486	NM_001031799	RasGEF domain family, member 1C
LGR5	0.491	NM_003667	leucine-rich repeat-containing G protein-coupled receptor 5
HSH2D	0.493	NM_032855	hematopoietic SH2 domain containing
**Group C**			
TLR4	0.099	NM_138554	toll-like receptor 4
TSSK2	0.421	NM_053006	testis-specific serine kinase 2
ADRB2	0.475	NM_000024	adrenergic, β-2-, receptor, surface
FLRT2	0.400	NM_013231	fibronectin leucine rich transmembrane protein 2
**Group D**			
E2F2	0.150	NM_004091	E2F transcription factor 2
ADRA2A	0.193	NM_000681	adrenergic, α-2A-, receptor
APLN	0.243	NM_017413	apelin, AGTRL1 ligand
REEP1	0.244	NM_022912	receptor accessory protein 1
ARHGAP26	0.252	NM_015071	Rho GTPase activating protein 26
UHRF1	0.259	NM_013282	ubiquitin-like, containing PHD and RING finger domains, 1
ZNF367	0.261	NM_153695	zinc finger protein 367
POU5F1	0.267	NM_203289	POU domain, class 5, transcription factor 1
RGS4	0.269	NM_005613	regulator of G-protein signalling 4
RHOJ	0.269	NM_020663	ras homolog gene family, member J
MCF2	0.270	NM_005369	MCF.2 cell line derived transforming sequence
CHRNA5	0.276	NM_000745	cholinergic receptor, nicotinic, α 5
GPR115	0.285	NM_153838	G protein-coupled receptor 115
SORCS3	0.286	NM_014978	sortilin-related VPS10 domain containing receptor 3
RBM14	0.287	NM_006328	RNA binding motif protein 14
PDE4B	0.298	NM_001037339	phosphodiesterase 4B, cAMP-specific (phosphodiesterase E4 dunce homolog, Drosophila)
PIK3CG	0.310	NM_002649	phosphoinositide-3-kinase, catalytic, γ polypeptide
RGPD2	0.311	NM_001024457	RANBP2-like and GRIP domain containing 2
TP53RK	0.314	NM_033550	TP53 regulating kinase
MAP2K6	0.320	NM_002758	mitogen-activated protein kinase kinase 6
TP73	0.330	NM_005427	tumor protein p73
GPR63	0.338	NM_030784	G protein-coupled receptor 63
FST	0.340	NM_006350	follistatin
MPP4	0.347	NM_033066	membrane protein, palmitoylated 4 (MAGUK p55 subfamily member 4)
PDE4D	0.350	NM_006203	phosphodiesterase 4D, cAMP-specific (phosphodiesterase E3 dunce homolog, Drosophila)
ANXA10	0.355	NM_007193	annexin A10
**Group D**			
RBL1	0.355	NM_002895	retinoblastoma-like 1 (p107)
KIT	0.360	NM_000222	v-kit Hardy-Zuckerman 4 feline sarcoma viral oncogene homolog
PBX1	0.368	NM_002585	pre-B-cell leukemia transcription factor 1
MTUS1	0.371	NM_001001924	mitochondrial tumor suppressor 1
RORB	0.386	NM_006914	RAR-related orphan receptor B
LHX6	0.389	NM_014368	LIM homeobox 6
PAQR4	0.392	NM_152341	progestin and adipoQ receptor family member IV
ABRA	0.394	NM_139166	actin-binding Rho activating protein
GDAP1	0.396	NM_018972	ganglioside-induced differentiation-associated protein 1
C1QTNF2	0.399	NM_031908	C1q and tumor necrosis factor related protein 2
CMTM1	0.400	NM_181289	CKLF-like MARVEL transmembrane domain containing 1
MLR1	0.404	NM_153686	transcription factor MLR1
TSPAN8	0.405	NM_004616	tetraspanin 8
SH2D4B	0.406	NM_207372	SH2 domain containing 4B
E2F1	0.406	NM_005225	E2F transcription factor 1
VANGL1	0.411	NM_138959	vang-like 1 (van gogh, Drosophila)
DUSP6	0.415	NM_001946	dual specificity phosphatase 6
FZD3	0.416	NM_017412	frizzled homolog 3 (Drosophila)
PPARGC1A	0.417	NM_013261	peroxisome proliferative activated receptor, γ, coactivator 1, α
HOXB7	0.419	NM_004502	homeobox B7
PTGER2	0.420	NM_000956	prostaglandin E receptor 2 (subtype EP2), 53 kDa
NGEF	0.421	NM_019850	neuronal guanine nucleotide exchange factor
FGF18	0.421	NM_033649	fibroblast growth factor 18
LOC653528	0.425	XM_927910	similar to Teratocarcinoma-derived growth factor 2 (Epidermal growth factor-like cripto protein CR3) (Cripto-3 growth factor)
OR4N2	0.426	NM_001004723	olfactory receptor, family 4, subfamily N, member 2
NKX6-2	0.429	NM_177400	NK6 transcription factor related, locus 2 (Drosophila)
NFKBIL2	0.431	NM_013432	nuclear factor of κ light polypeptide gene enhancer in B-cells inhibitor-like 2
PTPN22	0.431	NM_012411	protein tyrosine phosphatase, non-receptor type 22 (lymphoid)
LOC392269	0.432	XM_928112	similar to Transcription factor SOX-2
MAL2	0.432	NM_052886	mal, T-cell differentiation protein 2
SELPLG	0.434	NM_003006	selectin P ligand
GPR177	0.434	NM_001002292	G protein-coupled receptor 177
NCOA5	0.437	NM_020967	nuclear receptor coactivator 5
RIF1	0.437	NM_018151	RAP1 interacting factor homolog (yeast)
GPR3	0.439	NM_005281	G protein-coupled receptor 3
CDC14A	0.439	NM_003672	CDC14 cell division cycle 14 homolog A (*S. cerevisiae*)
RP3-509I19.5	0.444	XM_294019	similar to ECT2 protein (Epithelial cell transforming sequence 2 oncogene)
ADORA1	0.444	NM_000674	adenosine A1 receptor
PTCH	0.446	NM_000264	patched homolog (Drosophila)
TCF21	0.446	NM_003206	transcription factor 21
SPRY4	0.448	NM_030964	sprouty homolog 4 (Drosophila)
CBX2	0.450	NM_005189	chromobox homolog 2 (Pc class homolog, Drosophila)
OR6C74	0.451	NM_001005490	olfactory receptor, family 6, subfamily C, member 74
**Group D**			
CXCL14	0.452	NM_004887	chemokine (C-X-C motif) ligand 14
CUBN	0.453	NM_001081	cubilin (intrinsic factor-cobalamin receptor)
NRG2	0.457	NM_013985	neuregulin 2
SGIP1	0.457	NM_032291	SH3-domain GRB2-like (endophilin) interacting protein 1
GNGT2	0.457	NM_031498	guanine nucleotide binding protein (G protein), γ transducing activity polypeptide 2
EBF	0.458	NM_024007	early B-cell factor
ACVR1C	0.458	NM_145259	activin A receptor, type IC
PHTF2	0.458	NM_020432	putative homeodomain transcription factor 2
RASSF1	0.460	NM_007182	Ras association (RalGDS/AF-6) domain family 1
GPR109A	0.462	NM_177551	G protein-coupled receptor 109A
TSHR	0.463	NM_000369	thyroid stimulating hormone receptor
SIM2	0.468	NM_009586	single-minded homolog 2 (Drosophila)
GABRA6	0.469	NM_000811	γ-aminobutyric acid (GABA) A receptor, alpha 6
LAT2	0.469	NM_032464	linker for activation of T cells family, member 2
PHKG1	0.472	NM_006213	phosphorylase kinase, γ 1 (muscle)
RGPD4	0.473	XM_496581	RANBP2-like and GRIP domain containing 4
NKD1	0.474	NM_033119	naked cuticle homolog 1 (Drosophila)
ZNF588	0.475	NM_001013746	zinc finger protein 588
SH3TC2	0.476	NM_024577	SH3 domain and tetratricopeptide repeats 2
FZD1	0.478	NM_003505	frizzled homolog 1 (Drosophila)
PKMYT1	0.478	NM_004203	protein kinase, membrane associated tyrosine/threonine 1
DUSP4	0.480	NM_001394	dual specificity phosphatase 4
WDR4	0.480	NM_018669	WD repeat domain 4
WDR76	0.481	NM_024908	WD repeat domain 76
WDHD1	0.483	NM_001008396	WD repeat and HMG-box DNA binding protein 1
HOXA7	0.485	NM_006896	homeobox A7
WDR69	0.486	NM_178821	WD repeat domain 69
TFAP2C	0.487	NM_003222	transcription factor AP-2 γ (activating enhancer binding protein 2 γ)
CDGAP	0.488	NM_020754	Cdc42 GTPase-activating protein
RPIB9	0.491	NM_138290	Rap2-binding protein 9
IFNAR1	0.493	NM_000629	interferon (α, β and ω) receptor 1
POU3F2	0.493	NM_005604	POU domain, class 3, transcription factor 2
LOC402279	0.497	XM_377945	similar to glutamate receptor, metabotropic 8
EYA4	0.498	NM_004100	eyes absent homolog 4 (Drosophila)
ISL1	0.498	NM_002202	ISL1 transcription factor, LIM/homeodomain, (islet-1)
SIRPD	0.499	NM_178460	signal-regulatory protein δ
NEDD9	0.499	NM_182966	neural precursor cell expressed, developmentally down-regulated 9
TLR3	0.500 ^#^	NM_003265	toll-like receptor 3
Group E			
RAPSN	0.354	NM_005055	receptor-associated protein of the synapse, 43 kDa
GRAP	0.363	NM_006613	GRB2-related adaptor protein
CD48	0.410	NM_001778	CD48 molecule
LOC642966	0.428	XM_926351	similar to olfactory receptor 139
SALL1	0.437	NM_002968	sal-like 1 (Drosophila)
**Group E**			
GLIS1	0.438	NM_147193	GLIS family zinc finger 1
FOLR1	0.471	NM_016725	folate receptor 1 (adult)
NRG4	0.482	NM_138573	neuregulin 4
Group F			
TACR1	0.408	NM_015727	tachykinin receptor 1
NHLH1	0.414	NM_005598	nescient helix loop helix 1
NF2	0.420	NM_181825	neurofibromin 2 (bilateral acoustic neuroma)
LOC440607	0.427	NM_001004340	Fc-γ receptor I B2
MAF	0.443	NM_001031804	v-maf musculoaponeurotic fibrosarcoma oncogene homolog (avian)
ZNF160	0.449	NM_033288	zinc finger protein 160
DUSP2	0.454	NM_004418	dual specificity phosphatase 2
SOCS1	0.456	NM_003745	suppressor of cytokine signaling 1
CHRNA3	0.471	NM_000743	cholinergic receptor, nicotinic, α 3
CRLF2	0.481	NM_022148	cytokine receptor-like factor 2
MRAP	0.487	NM_178817	melanocortin 2 receptor accessory protein
RPIP8	0.491	NM_006695	RaP2 interacting protein 8
**Group G**			
CLEC4G	0.198	NM_198492	C-type lectin superfamily 4, member G
FSTL4	0.246	NM_015082	follistatin-like 4
RAB6C	0.282	NM_032144	RAB6C, member RAS oncogene family
CSF3	0.295	NM_172220	colony stimulating factor 3 (granulocyte)
OR2T34	0.301	NM_001001821	olfactory receptor, family 2, subfamily T, member 34
RRP22	0.304	NM_001007279	RAS-related on chromosome 22
UTF1	0.305	NM_003577	undifferentiated embryonic cell transcription factor 1
CHRND	0.306	NM_000751	cholinergic receptor, nicotinic, δ
GPR6	0.316	NM_005284	G protein-coupled receptor 6
ANGPTL6	0.324	NM_031917	angiopoietin-like 6
OR2M7	0.346	NM_001004691	olfactory receptor, family 2, subfamily M, member 7
OR10P1	0.356	NM_206899	olfactory receptor, family 10, subfamily P, member 1
FOXD3	0.364	NM_012183	forkhead box D3
ZAP70	0.377	NM_207519	ζ-chain (TCR) associated protein kinase 70 kDa
PTGER3	0.392	NM_000957	prostaglandin E receptor 3 (subtype EP3)
CDX4	0.395	NM_005193	caudal type homeobox transcription factor 4
TBX21	0.405	NM_013351	T-box 21
TAS2R13	0.408	NM_023920	taste receptor, type 2, member 13
IL17RE	0.414	NM_153482	interleukin 17 receptor E
PRDM9	0.415	NM_020227	PR domain containing 9
CXCL12	0.422	NM_199168	chemokine (C-X-C motif) ligand 12 (stromal cell-derived factor 1)
LILRA4	0.430	NM_012276	leukocyte immunoglobulin-like receptor, subfamily A (with TM domain), member 4
LOC642506	0.433	XM_926003	similar to double homeobox 4c
NEUROD6	0.435	NM_022728	neurogenic differentiation 6
KLF14	0.438	NM_138693	Kruppel-like factor 14
TFAP2E	0.439	NM_178548	transcription factor AP-2 ε (activating enhancer binding protein 2 ε)
**Group G**			
CCL1	0.439	NM_002981	chemokine (C-C motif) ligand 1
VAV3	0.439	NM_006113	vav 3 oncogene
IRS3L	0.444	XM_498229	insulin receptor substrate 3-like
GPR81	0.445	NM_032554	G protein-coupled receptor 81
GPR32	0.445	NM_001506	G protein-coupled receptor 32
GDF7	0.446	NM_182828	growth differentiation factor 7
WDR42C	0.447	XM_293354	WD repeat domain 42C
LOC619207	0.454	XM_927516	scavenger receptor protein family member
FOLR1	0.457	NM_016724	folate receptor 1 (adult)
ADRA1D	0.457	NM_000678	adrenergic, α-1D-, receptor
IL12RB2	0.459	NM_001559	interleukin 12 receptor, β 2
GRIN1	0.460	NM_007327	glutamate receptor, ionotropic, *N*-methyl D-aspartate 1
SHC2	0.461	XM_375550	SHC (Src homology 2 domain containing) transforming protein 2
RAXL1	0.464	NM_032753	retina and anterior neural fold homeobox like 1
CAMK2B	0.472	NM_172084	calcium/calmodulin-dependent protein kinase (CaM kinase) II β
CCL15	0.473	NM_004167	chemokine (C-C motif) ligand 15
FSHR	0.474	NM_000145	follicle stimulating hormone receptor
WDR40B	0.478	NM_178470	WD repeat domain 40B
MAFB	0.482	NM_005461	v-maf musculoaponeurotic fibrosarcoma oncogene homolog B (avian)
TPRX1	0.484	NM_198479	tetra-peptide repeat homeobox 1
FLT1	0.487	NM_002019	fms-related tyrosine kinase 1 (vascular endothelial growth factor/vascular permeability factor receptor)
OLIG2	0.488	NM_005806	oligodendrocyte lineage transcription factor 2
TBXA2R	0.490	NM_001060	thromboxane A2 receptor
SSTR5	0.490	NM_001053	somatostatin receptor 5
MYOG	0.491	NM_002479	myogenin (myogenic factor 4)
OR2AG1	0.492	NM_001004489	olfactory receptor, family 2, subfamily AG, member 1
FOXD4L1	0.495	NM_012184	forkhead box D4-like 1
PSPN	0.495	NM_004158	persephin
PJCG6	0.496	NM_001040066	similar to olfactory receptor 873
TSHR	0.499	NM_001018036	thyroid stimulating hormone receptor

* ratio of the expression in cells cultured at pH 6.7 after 24, 5, and 2 h culture to those at pH 7.5 in groups A to D, E to F, and G, respectively; ^#^ 0.499857.

### 3.4. The Gene Expressions in Various Cells

To confirm whether or not the gene expression pattern observed in mesothelioma cells is specific to these cells, we selected six genes, IL-32, TNFRSF9, AREG, ERBB3, ATP6V0D2, and DMGDH whose expressions were observed to increase more than three-fold at acidic pH using a microarray, and examined their expressions in various cells. IL-32 has been reported to be a cytokine, but its function remains unclear. TNFRSF9, AREG, and ERBB3 have been reported to be a receptor, growth factor, and oncogene product, respectively. ATP6V0D2 is one subunit of ATPase which has a role in pH regulation. DMGDH is a mitochondrial matrix enzyme.

One problem in the measurement of mRNA was determining which gene was available as a control gene. A housekeeping gene such as GAPDH has been used generally until now. The previous report from our group showed that the amount of 18S rRNA was constant at both acidic and alkaline pH in human T cells [15]. The amount of 18S rRNA in mesothelioma cells did not vary as pH changed (data not shown). Based on these data, 18S rRNA was used as a control RNA in this study. The amount of ribosomes per cell was approximately 4 × 10^6^ [25]. The copy number of mRNA per cell can be estimated using this number. 

IL-32, AREG, ERBB3, ATP6V0D2, and DMGDH genes showed increased expression in TE-11 cells after 24 h culture at acidic pH (Figure 1). These data were in agreement with the DNA array data. In contrast, the expression of TNFRSF9 did not increase under acidic pH in TE-11 (Figure 1).

The expression of IL-32 was increased at acidic pH in HT-29, HepG2, and BxPC3. HT-29 cells showed increased expressions of ERBB3, ATP6V0D2, and DMGDH at acidic pH, but the expressions of AREG and TNFRSF9 decreased. The expression of ATP6V0D2 was decreased by acidosis in BxPC3. The expressions of AREG, ERBB3, and ATP6V0D2 were not affected by pH in HepG2. These results suggested that genes whose expression is stimulated at acidic pH are different in different cells.

## 4. Discussion

The effect of acidosis on gene expression has been generally studied in medium without FBS until now. Cells are unable to proliferate under these conditions. In the present study, we used medium containing FBS which supported cell proliferation. We used medium without the addition of bicarbonate, because the medium pH was changed after the medium had been put into a CO_2_ incubator and the measurement of the exact pH value in the CO_2_ incubator was difficult. When the CO_2_ incubator is not used, the addition of bicarbonate is not necessary for cell proliferation. Bicarbonate is produced via metabolic processes, such as glycolysis and the citric acid cycle under aerobic conditions, and the production is enough for cell proliferation. In fact, all cell lines we used proliferated in medium without the addition of bicarbonate, and the proliferation rate was the same as that in medium with the addition of bicarbonate. The number of mesothelioma cells increased twofold during 2 days of incubation at pH 6.7 under our experimental conditions, but no proliferation was observed at pH 6.5 or less. We therefore used pH 6.7 medium under acidic conditions in this study.

Some diseased areas are acidified, but the acidification is less than 1 pH unit in many cases. Such a small change in pH has been thought to have little effect on mammalian cell functions until now. Our present data, however, clearly showed that acidification affects gene expression even if the pH change is small. Approximately 24,000 genes, about two-thirds of the mammalian genes, were analyzed in the present study, and 693 genes were up-regulated and 856 genes were down-regulated more than twofold at acidic pH in mesothelioma cells (Table 3, Table 6).

The expressions of 260 genes increased more than twofold in cells cultured at pH 6.7 for 2 h compared with pH 7.5. The expressions of 223 among the 260 genes decreased again after 24 h (Table 3). The physiological significance of the expression for a short time remains unclear. It is probably not due to the fluctuation of internal pH because the internal pH was decreased within 1 h after the acidic shift and then maintained at a constant level (data not shown). It has been generally accepted that the activation of the signal proteins increases rapidly after the stimulation and then decreases. It could be suggested that the expression levels of some genes for signal proteins decrease after the initial stimulation, although no direct evidence has yet been reported.

Our group found that the decrease in external pH below 7 changes the signal pathways, at least in part [11,12,15], and we identified a gene product that was essential for proliferation at acidic pH [13]. The present data showed that 84 genes for signaling were expressed more strongly after 24 h culture at acidic pH. The functions of the 78 genes whose expressions were up-regulated at acidic pH are unknown. It might be possible that some of these unidentified genes encode proteins for cellular signaling.

Since translational activities are different in different genes, the mRNA level is not proportional to the enzyme level. Therefore, all protein levels encoded by genes whose expression is affected by pH may be required for clarifying the signal pathways working at acidic pH. Furthermore, there are some genes whose expression is constitutive, but function is preferential at acidic pH. Lao *et al*. found CTIB to be essential for growth at acidic pH, but its expression was not affected by pH in the range from 6 to 8 [13,14,27]. p38 and ERK were activated strongly at acidic pH [12,15], but our present results showed no significant stimulation of their expression by acidosis. Identification of such proteins will be essential for improving our understanding of signal pathways operating under acidic diseased loci, and our present data could be useful for these studies as a database at the transcriptional level.

We found that different cytokines are expressed under different pH conditions (Table 4, Table 7). Especially IL-32 was found to express at a higher level at acidic pH in various cells. IL-32 was first identified in natural killer (NK) cells and IL-2 activated T cells [28], and was designated NK4. Since recombinant NK4 induced TNF-α production in human macrophages, it was assumed to have interleukin-like activity and hence was designated IL-32 [29]. Subsequent studies suggested that IL-32 is linked with pathological inflammation which often causes an acidic environment. Elevated IL-32 concentrations in synovial fluids and synovial tissues were demonstrated in rheumatoid arthritis but not in osteoarthritis patients [30,31]. Up-regulated IL-32 expression was also observed in the pancreatic ducts of chronic pancreatitis patients [32]. Taken together with our present data, IL-32 may be a factor that works under acidic conditions, but is not a cytokine specific to immune functions. IL-8, IL-15, and IL-16 were also up-regulated at acidic pH, and these interleukins may work in acidic diseased areas.

Our present data suggest that different signal pathways operate under different pH conditions. Why do mammalian cells have this multiplicity of signaling systems? The underlying mechanism is still unclear. Cytosolic pH changed with the change in extracellular pH, and the change in internal pH may affect protein activity because all proteins have pH-dependent activity. One possible explanation is that an enzyme having maximum activity at acidic pH works under acidic pH instead of the enzyme having maximum activity at alkaline pH. *E. coli* has multiple transport systems for sodium and potassium ions, and these systems work under different pH conditions [9,10]. Glycolysis was reported to increase in several tumors [1,2,3]. Only phosphoglycerate mutase 2 (muscle) was increased 2.03-fold at acidic pH (supplementary table), suggesting that other enzymes still work under acidic conditions without the elevation of transcription. Phosphoglycerate mutase 2 was reported to be a muscle-specific enzyme [33]. Since the muscles are often acidified, it can be argued that this enzyme works at acidic pH and the other isozyme does at alkaline pH.

The expressions of many receptor genes were affected by the pH change (Table 4, Table 7). Since receptors in the cytoplasmic membranes generally have a domain located outside the cells, the activity may be more sensitive to external acidosis compared with the cytosolic enzymes, and the gene expression of many receptors having an optimum activity at acidic pH may be stimulated by acidosis to compensate for the functional decline of receptors having an optimum activity at alkaline pH.

We used mesothelioma cells in the present study. Since the gene expression patterns were shown to be different in different cells, our present data may be applicable only to responses of mesothelioma cells. Analysis of the gene expressions in various cells, including non-tumor cells and normal tissues under acidic conditions will be essential for clarifying cell functions in acidic diseased areas.

## 5. Conclusions

Some diseased areas, such as cancer nests, inflammatory loci, and infarction areas, are acidified, but the acidification is less than 1 pH unit in many cases. Our present data clearly showed that acidification affects gene expression even if the pH change is small. Approximately 24,000 genes, about two-thirds of the mammalian genes, were analyzed using mesothelioma cells. The expressions of 693 genes were up-regulated more than twofold at acidic pH, and genes encoding proteins for signal pathways numbered 165 among the 693 genes. The expressions of 856 genes were down-regulated more than twofold at acidic pH, and 194 among the 856 genes encoded proteins for signal pathways.

## Supplementary Files

Supplementary File 1 Supplemental Table (XLS, 12326 KB)
